# Taking Some of the Mystery out of Host∶Virus Interactions

**DOI:** 10.1371/journal.ppat.1002033

**Published:** 2011-05-05

**Authors:** Karen-Beth G. Scholthof

**Affiliations:** Department of Plant Pathology and Microbiology, Texas A&M University, College Station, Texas, United States of America; The Fox Chase Cancer Center, United States of America

Plant viruses induce a spectrum of symptoms on infected plants, including mosaics (or mottles), ringspots, and local lesions. The mechanism of host∶virus interactions responsible for phenotypic changes has largely remained mysterious. Two papers in this issue of *PLoS Pathogens* [1], [2] have used overlapping, creative experimental approaches—reflecting their interests in the “plant side” or the “virus side” of the problem—to get at the mechanism of a brilliant yellow phenotype (Y-phenotype) observed in plants in one particular host∶virus interaction. The two groups use state-of-the art tools to demonstrate the utility of plant viruses for making fundamental discoveries in plant “omics”. This work, most of which was performed using crop plants, deserves the attention of plant biologists.

The best science often results in many avenues for future work and occasionally opens up new fields of inquiry. The papers published by the research groups of Chikara Masuta [2] and Ming-Bo Wang [1] are exemplars of the promise that robust technical development holds for solving the most basic question in plant pathology: what causes disease? Specifically, in the two papers under discussion, a 339-nucleotide (nt) satellite RNA (Y-satRNA) of *Cucumber mosaic virus* (CMV) was used to examine the cause of the Y-phenotype on tobacco. CMV is a single-stranded, positive-sense RNA virus with three genome components. The host range of CMV is estimated to extend to more than 1,200 species of plants in 100 families, resulting in ongoing, devastating epidemics worldwide in economically important crops [3], [4]. CMV, the helper virus, supports the replication, encapsidation, and movement of a plethora of satRNAs, some of which worsen symptoms and cause severe effects on yield. Aphids or mechanical transmission move encapsidated CMV and its satRNAs from infected to healthy plants.

In 1960, a strain of CMV was described that induced a Y-phenotype on 14 *Nicotiana* species, including *Nicotiana tabacum* (tobacco) [5]. The presence of a satRNA was shown in 1977, followed in 1981 by the definitive identification of a unique RNA species named Y-satRNA [6], [7]. Tobacco plants infected with CMV alone remained green, but the mixed infection of CMV+Y-satRNA induced the Y-phenotype. The ultrastructure of the chloroplasts, lipid content and composition of the membranes, and stromal proteins were similar in plants infected with CMV alone or with CMV+Y-satRNA. However, total thylakoid protein accumulation and photosynthesis was decreased in the Y-satRNA infection [8]. In 1989, infectious cDNA constructs of Y-satRNA recapitulated the effect of inducing a “dramatic brilliant yellow mosaic” in tobacco co-infected with CMV; in tomato this infection induced necrosis [9]. By introducing point mutations or insertions of a few nucleotides, and upon comparisons with other CMV satRNAs, a single nucleotide at position 325 was associated with necrosis on tomato. A pathogenicity region with predicted significant secondary structure was defined and later narrowed to 24-nts on the Y-satRNA. When *Nicotiana bigelovii* and *Nicotiana clevelandii*, described as susceptible (Y-phenotype) and resistant (green phenotype), respectively, were crossed, the F_1_ plants had a mixed phenotype when challenged with CMV+Y-satRNA [10]. Similar challenges on segregating F_2_ populations caused 1∶2∶1 yellow∶intermediate∶green phenotypes. This suggested an “incomplete dominant” trait as a result of a nuclear-encoded gene, which was confirmed with a synthetically created *N. tabacum* line (cv. Consolation 402), with the nuclear genome of *N. tabacum* (Y-phenotype) and the hereditary cytosolic elements of *Nicotiana debneyi* (green phenotype). When challenged with CMV+Y-satRNA, the Y-phenotype was observed [10].

Yet the question remained: which nuclear gene(s) caused the Y-phenotype on tobacco during a CMV+Y-satRNA infection? In 1993, it was suggested that anti-sense inhibition (the forerunner to silencing) might be the mechanism, which could possibly be tested by “the analyses of enzymes involved in chlorophyll synthesis and their mRNA levels” [8]. Smith et al. have now shown this to be the case, by investigating which *Nicotiana* genes might be silenced by Y-satRNA [1]. Although no perfect match of the 24-nt pathogenicity region of Y-satRNA was found by BLAST, with some substitutions and further scanning in *Nicotiana* genome databases, a single host gene was identified: magnesium protoporphyrin chelatase subunit I (*ChlI*). Transgenic tobacco silenced for this gene developed the predicted mottled to Y-phenotype. Furthermore, the specificity of siRNA-mediated RISC cleavage was primarily restricted to three nucleotides within the 22-nt “target” site of the *ChlI* mRNA. Using next-generation sequencing, it was shown that 25% of siRNA was of Y-satRNA origin and that several thousand reads mapped primarily to the 5′-end nts 178, 180, and 181 on the Y-satRNA, midway in the pathogenic 22-nt region. With this preliminary evidence, Smith et al. returned to the plant to alter *ChlI* or satRNA sequences to evaluate the “host range” and stringency of the silencing effects, scored by Y-phenotype. They were able to slow the rate of the yellow phenotype (or decreased chlorophyll) during the CMV+Y-satRNA infection by overexpressing *ChlI* transgenically in the infected plants.

The tobacco *ChlI* gene also was further subjected to mutagenesis (*mtCHLI*) to abolish interaction with Y-satRNA, and transformed into tobacco. CMV+Y-satRNA infection of these plants was normal, but instead of the Y-phenotype, the plants remained green, thus biologically confirming a Y-satRNA:*ChlI* interaction. Next, they used *N. tabacum* infected with CMV+Y-satRNA (Y-phenotype) and performed grafting with root and scion stocks of transgenic *mtCHLI* plants. Wang's group found that although the virus moved through all parts of the plant, the *mtCHLI* leaves remained green [1]. This technique also opens up new strategies for rapid screening of possible virus∶host interactions. Smith et al. identified two other CMV satRNAs that match to a glycolate oxidase, another protein that induces yellowing when down-regulated, and suggested that similar gene discoveries could be made with viroid sequences using BLAST tools [1]. As this work continues, their strategy could allow for further rapid fine mutation analyses of Y-satRNA to determine its effects on chlorophyll biosynthesis and aid in the study of orthologous *ChlI* genes in other crop plants infected with CMV. This might also open up new avenues for the use of *Nicotiana benthamiana*, a promiscuous host for plant virus infection, for the study of biochemical and physiological effects of viruses, using chlorosis or any other affected genes or pathways that have a phenotypic consequence.

For the “virus side” of the interaction, we have the paper of Shimura et al. [2]. They hypothesized that *ChlI* might be a gene candidate, based on reports that silencing *ChlI* in cotton and *Arabidopsis* elicited symptoms, reminiscent of the CMV+Y-satRNA on tobacco [2]. If Y-satRNA and *ChlI* mRNA had complementary nucleotide regions, then transgenic plants expressing an inverted repeat of double-stranded Y-satRNA (YsatIR) should trigger silencing, scored by the Y-phenotype. Instead, for unknown reasons, the YsatIR *N. benthamiana* plants developed only minor patchy chlorosis, not the hypothesized brilliant yellow phenotype.

Shimura et al. then used the transgenic YsatIR plants as a source of RNA for microarray analyses. In comparison to wild-type plants, 134 significantly down-regulated genes were identified, of which 31 were associated with the chlorophyll pathway or chloroplast development. Interestingly, the 22-nt yellow phenotype region of Y-satRNA was found to align with a *ChlI* gene from the microarray. Revisiting the YsatIR plants with a candidate gene, Shimura et al. found that *ChlI* mRNA was indeed down-regulated, and they further confirmed their findings in *N. tabacum* plants and protoplasts with infections of CMV+Y-sat RNA, as well as with a 150-bp region of *ChlI* transcribed from a CMV gene vector. Significantly, and as predicted, three other CMV satRNAs (which did not induce the Y-phenotype) did not affect *ChlI* mRNA accumulation. Of the 31 candidate genes, chlorophyll a/b binding protein 3 (*CAB3*) also was decreased in CMV+Y-satRNA infections. As *ChlI* is a critical enzyme for chlorophyll biosynthesis, its perturbation likely affects the regulation of photosynthetic genes such as *CAB3*, something that was later confirmed using 2-D protein gels [2].

Extending their studies to other hosts, Shimura et al. show that the *ChlI* gene of pepper plants and the 22-nt Y-satRNA have perfect complementarity, and following virus infection, *ChlI* gene silencing and the Y-phenotype were observed. In *Arabidopsis* and tomato, a Y-phenotype was induced only when the Y-satRNA was mutated for the exact complementary sequence with the respective host *ChlI* genes. In contrast, these mutants no longer induced the bright Y-phenotype on tobacco and *ChlI* mRNA accumulation was not as perturbed. Interestingly, in tomato the Y-satRNA mutant quickly restored itself to the original RNA sequence. This provides several lines of evidence that there is an interaction between Y-satRNA and *ChlI* that triggers the Y-phenotype. From deep sequencing, Shimura et al. confirmed the presence of 21- and 22-nt siRNAs specific to the Y-satRNA in (+) and (−) sense orientations, but much lower amounts of *ChlI* siRNAs, likely due to the high levels of satRNA accumulation during a virus infection. Both groups analyzed the 5′-cleavage ends of *ChlI* siRNAs, which mapped to the middle of the 22-nt Y-satRNA complementary region using 5′-RACE. Shimura et al. took the additional innovative approach of using a GFP construct with the 22-nt region from *ChlI* inserted into the 3′-UTR. When agroinfiltrated into *N. benthamiana* plants infected with CMV+Y-satRNA (leading to Y-phenotype and reduced *ChlI*), GFP expression (fluorescence, mRNA, and protein) was quenched much more than when GPF alone was agroinfiltated into the same leaves, providing another line of evidence that the 22-nt Y-satRNA sequence was necessary and sufficient for site-directed silencing of the GFP marker [2].

Some *Nicotiana* species remain green when infected with CMV+Y-satRNA [5], suggesting slight variations of the *ChlI* gene sequence, especially within the region that is associated with the Y-phenotype. Both groups investigated this possibility. Analysis of *ChlI* sequences from four additional species was carried out and it was found that the Y-phenotype correlated with the degree of *ChlI* sequence match to the complementary 22-nt Y-satRNA region and was further confirmed by silencing concomitant with infection.

It is not an exaggeration to say that plants determine our future. And a crucial aspect of plant biology that sustains life on this planet is photosynthesis, which fixes CO_2_ into carbohydrates in the presence of sunlight and water. A first critical step, which sets the pathway to the synthesis of chlorophyll a and chlorophyll b, is the insertion of Mg^2+^ into the precursor protein protoporphyrin IX (PpIX). The enzyme for this reaction is a magnesium chelatase (*ChlI*), one of three proteins used to form a functional complex. An alternate route for PpIX is the protoheme that involves a Fe-chelatase, an enzyme that is conserved across taxonomic kingdoms, and which catalyzes the insertion of Fe^2+^ into PpIX [11], [12]. Since *ChlI* is a key regulator in the tetrapyrrole biosynthetic pathways leading to chlorophyll biogenesis, it is not surprising that remarkable conservation of both polypeptide sequence and function is maintained between orthologous *ChlI* genes in photosynthetic bacteria and plants [13], [14].

Since *ChlI* is nuclear encoded, this opens up ideas for further investigation, especially in light of recent findings of nuclear posttranscriptional gene silencing (PTGS), with evidence that 22-nt RNAs and some DICER-associated proteins accumulate in the nucleus [15]. That the silencing of *ChlI* is not 100%, as indicated by the data from both papers, exposes opportunities for further dissection of the mechanisms of silencing. For example, *ChlI* transcripts accumulate in the nucleus and cytosol. Is silencing occurring primarily in the cytosol or is the satRNA triggering nuclear PTGS? Since siRNAs traffic to the nucleus to drive RNA-dependent DNA-methylation, it will be interesting to determine if there is concomitant DNA methylation of the *ChlI* locus and whether it is stably heritable to subsequent generations. If this is the case, satRNAs might be deployed for modulation of protein accumulation, or modified to single out pre-mRNA sequences of target genes (such as *ChlI*), or to establish if silencing can be made stronger by degrading both nuclear and cytosolic forms of the target RNAs. This could provide new approaches and designs to use virus-based gene therapy for crop plant improvement.

It is known that *ChlI* expression increases in etiolated leaves as they become green and is constitutively expressed in mature leaves. Since *ChlI* mutants in *Arabidopsis*, maize, and tobacco display age-related pale green to yellow phenotypes [14], [16], [17], perhaps CMV+Y-satRNA could be inoculated to different ages of plants to study temporal gene expression, especially as related to plant developmental biology. The investigation of the accumulation and localization of the *ChI1* protein in future studies will bring together the long-term goals of plant pathologists—to understand more about the physiological aspects of host∶virus interactions in order to improve crop yields and control virus infections.

It is tempting to speculate that the significant decrease in *ChlI* gene expression due to silencing may result in increased pools of protoheme, the other branch for the tetrapyrrole pathway, and perhaps increase cytochrome accumulation or affect the biosynthesis of precursors such as 5-aminolevulinic acid. Both papers show downstream expression of chlorophyll a/b accumulation is reduced [1], [2]. From this, abolishment of *ChlI* due to Y-satRNA may also reduce the feedback mechanism to synthesize new chlorophyll, increase heme production, and affect signaling of nuclear gene expression [18]. Interestingly, since heme has been shown to activate toll-like receptor 4, which enhances the host inflammatory response [19], one wonders if analogous innate immunity-like effects are associated with Y-satRNA-infected plants. And, there could be some evolutionary advantage of the Y-phenotype to the virus, as infected plants attract many aphids, which, in turn, acquire and transmit CMV+Y-satRNA during feeding [2]. Nevertheless, modifications of satRNA or plant viruses in general, and virus gene vectors may prove quite useful in functional analyses of critical enzyme pathways, and may add to the collections of mutants that are available for model plant systems, as well as providing opportunities to create new mutants in other plant species.

Both papers identify millions of sRNAs, with upwards of 25% of the total representing satRNA. That a 21–24-nt region on the satRNA is complementary to the *ChlI* gene brings up the idea that satRNAs are derived from the host. Perhaps during virus replication, with an error-prone replicase and strand switching, satellite RNAs occasionally emerge with 5′- and 3′-termini of the helper virus and a patchwork of host RNAs. Many satRNAs ameliorate symptoms or reduce the accumulation of the helper virus, which may be another example of the Red Queen in action. The Y-satRNA probably has a few more lessons to teach us in the coming years.

My intent has been to convey that the high caliber work demonstrated by these researchers will have far ranging effects in plant biology and plant pathology. Several recent scientific advances have made this work possible, including development of plant viruses as gene vectors, microarray and next-generation sequencing technologies, gene silencing as a tool to study host genes, and the use of model plant systems. It seems we are at the dawn of mechanistic plant virology studies. Following more than 40 years of research on the CMV satRNAs, we now have a new template for thinking about the origin and evolution of viruses and to simultaneously translate these primary technological advantages to control crop losses due to virus infections.

## References

[1] Smith NA, Eamens AL, Wang M-B (2011). Viral siRNAs target host genes to mediate disease symptoms in plants.. PLoS Pathog.

[2] Shimura H, Pantaleo V, Ishihara T, Myojo N, Burgyán Jz (2011). A viral satellite RNA induces yellow symptoms on tobacco by targeting a gene involved in chlorophyll biosynthesis using the RNA silencing machinery.. PLoS Pathog.

[3] Zitter TA, Murphy JF (2009). Cucumber mosaic virus.. http://www.apsnet.org/edcenter/intropp/lessons/viruses/Pages/Cucumbermosaic.aspx.

[4] Gallitelli D (2000). The ecology of cucumber mosaic virus and sustainable agriculture.. Virus Res.

[5] Tomaru K, Hidaka Z (1960). Strains of cucumber mosaic virus isolated from tobacco plants. III. A yellow strain.. Bull Hatano Tobacco Exp Sta [Japanese, English abstract].

[6] Takanami Y (1981). A striking change in symtpoms on cucumber mosaic virus-infected tobacco plants induced by satellite RNA.. Virology.

[7] Takanami Y, Kubo S, Imaizumi S (1977). Synthesis of single- and double-stranded cucumber mosaic virus RNAs in tobacco mesophyll protoplasts.. Virology.

[8] Masuta C, Suzuki M, Matsuzaki T, Honda I, Kuwata S (1993). Bright yellow chlorosis by cucumber mosaic virus Y satellite RNA is specifically induced without severe chloroplast damage.. Physiol Molec Plant Pathol.

[9] Masuta C, Takanami Y (1989). Determination of sequence and structural requirements for pathogenicity of a cucumber mosaic virus satellite RNA (Y-satRNA).. Plant Cell.

[10] Masuta C, Suzuki M, Kuwata S, Takanami Y, Koiwai A (1993). Yellow mosaic symptoms induced by Y satellite RNA of cucumber mosaic virus is regulated by a single incompletely dominant gene in wild Nicotiana species.. Phytopathology.

[11] Mochizuki N, Tanaka R, Grimm B, Masuda T, Moulin M (2010). The cell biology of tetrapyrroles: a life and death struggle.. Trends Plant Sci.

[12] Tanaka R, Tanaka A (2007). Tetrapyrrole biosynthesis in higher plants.. Annu Rev Plant Biol.

[13] Bollivar D (2006). Recent advances in chlorophyll biosynthesis.. Photosynth Res.

[14] Sawers R, Farmer P, Moffett P, Brutnell T (2006). In planta transient expression as a system for genetic and biochemical analyses of chlorophyll biosynthesis.. Plant Methods.

[15] Hoffer P, Ivashuta S, Pontes O, Vitins A, Pikaard C (2011). Posttranscriptional gene silencing in nuclei.. Proc Natl Acad Sci USA.

[16] Ruairidh JH, Sawers JV, Farmer PR, Bussey RR, Olsefski G (2006). The maize *Oil Yellow1* (*Oy1*) gene encodes the I subunit of magnesium chelatase.. Plant Mol Biol.

[17] Huang Y-S, Li H-m (2009). Arabidopsis CHLI2 can substitute for CHLI1.. Plant Physiol.

[18] Ikegami A, Yoshimura N, Motohashi K, Takahashi S, Romano PGN (2007). The CHLI1 Subunit of Arabidopsis thaliana magnesium chelatase is a target protein of the chloroplast thioredoxin.. J Biol Chem.

[19] Figueiredo RT, Fernandez PL, Mourao-Sa DS, Porto BN, Dutra FF (2007). Characterization of heme as activator of toll-like receptor 4.. J Biol Chem.

